# A steganalysis-based approach to comprehensive identification and characterization of functional regulatory elements

**DOI:** 10.1186/gb-2006-7-6-r49

**Published:** 2006-06-20

**Authors:** Guandong Wang, Weixiong Zhang

**Affiliations:** 1 Department of Computer Science and Engineering, Washington University, St. Louis, MO 63130, USA; 2 Department of Genetics, Washington University, St. Louis, MO 63130, USA

## Abstract

The comprehensive identification of *cis*-regulatory elements on a genome scale is a challenging problem. We develop a novel, steganalysis-based approach for genome-wide motif finding, called WordSpy, by viewing regulatory regions as a stegoscript with *cis*-elements embedded in 'background' sequences. We apply WordSpy to the promoters of cell-cycle-related genes of *Saccharomyces cerevisiae *and *Arabidopsis thaliana*, identifying all known cell-cycle motifs with high ranking. WordSpy can discover a complete set of *cis*-elements and facilitate the systematic study of regulatory networks.

## Abstract 2

WordSpy, a novel, steganalysis-based approach for genome-wide motif-finding is described and applied to yeast and *Arabidopsis *promoters, identifying cell-cycle motifs.

## Background

The comprehensive identification and characterization of short functional sequence elements has become increasingly important as we begin to elucidate transcriptional regulation on a large scale. Transcriptional regulation involves a complex molecular network. The interaction of transcription factors (TFs) and *cis*-acting DNA elements determines the expression levels of different genes under various environmental conditions [1]. Deciphering such a network is to infer regulatory rules that can properly explain the expressions of different genes with the regulatory elements in their promoters and the presence of TFs [2,3]. Therefore, a complete set of regulatory elements is essential for systematic analysis of transcriptional regulation networks on a genome-wide scale.

The discovery of *cis*-regulatory elements in a genome has been a challenging problem for decades. Most widely applied approaches first cluster genes into small groups with similar expression profiles or similar biological functions, and then search for common short sequences (or motifs) in the regulatory regions of the genes in a group. This is based on the assumption that coexpressed genes are more likely to be co-regulated. Many efficient algorithms, including multiple local alignment-based [4-7], word enumeration-based [8], and dictionary-based [9], have been developed to search for statistically significant motifs from a small number of sequences. Despite the success of these methods, this approach has noticeable limitations. Computational gene clustering is often inaccurate and subjective, in terms of what similarity measure to use and how many clusters to form. Importantly, many genes belonging to a common pathway may have similar expression patterns, but are not regulated by the same TFs. Furthermore, transcriptional regulation is combinatorial [1], in that a regulatory element needs to combine with various others to function under different conditions. This means that the same motif may appear in the promoters of genes that express or function differently. Therefore, clustering genes into small sets may split the genes containing a particular set of motifs into different clusters, which makes it difficult, if not impossible, to find all regulatory elements [10].

In recent years, comparative genome analysis has been successfully applied to the discovery of regulatory motifs [11,12]. Taking advantage of sequence conservation in related species, this approach can effectively identify regulatory elements on a genome scale without any prior knowledge of co-regulation or gene function. This approach is limited in some situations, however. First, the species considered in a comparative analysis must be properly diversified evolutionarily. They must be evolutionarily separated long enough to allow nonfunctional elements to diverge. On the other hand, they must not be evolutionarily too far apart from one another so that functional elements remain conserved. For many applications, not many such genomes are available. Second and more important, there exist species-specific regulatory elements, which a comparative genomic method can hardly detect.

In this paper we propose a novel genome-wide approach to comprehensively identify regulatory elements from a single genome. Instead of clustering genes into groups, we use all the genes of interest together - for instance, the genes related to a particular biological process such as the cell cycle or the genes responding to a particular stress condition. In this approach, we first search for statistically over-represented motifs as completely as possible. We then use additional information, such as the coherency of expression profiles of genes containing a motif and the specificity of a motif to target genes, in order to evaluate the biological relevance of the extracted motifs so as to find truly functional regulatory elements.

We view this genome-wide motif-finding problem from a perspective of steganography and steganalysis. Steganography is a technique for concealing the existence of information by embedding the messages to be protected in a covertext to create a 'stegoscript' [13]. Steganalysis is the deciphering of a stegoscript by discovering the hidden message [13]. In this approach, we consider the regulatory regions of a genome as though they constituted a stegoscript with over-represented words (that is, regulatory elements) embedded in a covertext (that is, 'background' genomic sequences). We then model the stegoscript with a statistical model - a hidden Markov model [14] - consisting of a dictionary of motifs and a grammar. We progressively learn a series of models that are most likely to have generated the script. The final model is then used to decipher the stegoscript as well as to extract over-represented motifs. On the basis of this novel viewpoint, we have developed an efficient genome-wide motif-finding algorithm called WordSpy that can discover a large number of motifs from a large collection of regulatory sequences. Note that our technical approach of using a dictionary is inspired by the work of Bussemaker *et al*. [15], in which they introduced innovative ideas of segmenting sequences into words and building a dictionary of words from the sequences.

Our WordSpy method has several salient properties. First of all, by statistically modeling the regulatory regions as stegoscripts, WordSpy aims to discover a complete set of significant motifs. Therefore, instead of being trapped by some pseudo-motifs, for example, over-represented repeats, WordSpy includes them in its model, making it less vulnerable to spurious motifs. Second, WordSpy combines word counting and statistical modeling. It applies word counting to efficiently detect high-frequency words. It then enhances the representation of words by position weight matrices (PWMs) [16] to capture degenerate motifs. Third, WordSpy is able to detect discriminatory motifs that can be used to properly separate two sets of sequences. Finally, by incorporating gene-expression information and a genome-wide specificity analysis, we augment the basic algorithm in order to distinguish biologically relevant motifs from spurious ones, making the overall method practical for genome-wide identification of functional *cis*-regulatory elements, as we will demonstrate here.

We will first evaluate the method with an English stegoscript and 645 cell-cycle-related genes of *Saccharomyces cerevisiae*. We will then apply it to identify cell-cycle-related motifs from more than 1,000 genes in model plant, *Arabidopsis thaliana*. Furthermore, we will apply WordSpy as a discriminative motif-finding algorithm by incorporating TF location information - that is, chromatin immunoprecipitation DNA binding microarray (ChIP-chip) data - and build a dictionary of motifs for each known TF of budding yeast. Finally, we compare WordSpy with a set of existing methods on a benchmark that includes 56 well-curated sets of sequences and motifs in four species [17].

## Results and discussion

### Stegoscripts and the statistical model

The regulatory regions of a genome encode transcriptional regulatory information using regulatory elements embedded in background sequences. We can thus view the regulatory regions of the genes of interest as a stegoscript, which conceals the secret messages (*cis*-elements) with some covertext (background sequences). The hidden secret messages are typically more conserved and statistically over-represented than those in the covertext. This is particularly true for genomic regulatory sequences, where a small number of TFs regulate a large number of genes [1], making functional *cis*-elements over-represented.

Consider a set of regulatory sequences or a stegoscript *S *= (*S*_1_,*S*_2_,...,*S*_*q*_) where *S*_*i *_= (*S*_*i*1_*S*_*i*2_...) and *l*_*i *_is the length of the *i*th (*i *= 1, 2,..., *q*) sequence. Deciphering the script is to annotate the sequences with a series of substrings *χ *= (*x*_1_,*x*_2_,...,*x*_*t*_), where *x*_*j *_denotes the *j*th substring with length *l*(*x*_*j*_), which can be a background word or a functional element. In general, a stegoscript is a product of a grammar, by which all possible scripts in the language can be generated by successively rewriting strings according to a set of rules. Therefore, we model the stegoscript statistically. The model captures regulatory motifs and background words by a dictionary, and specifies how the motifs and words are used to form the stegoscript by a grammar. Given the statistical model, *χ *is just the optimal parse over *S *using the words in the dictionary.

**Figure 1:**
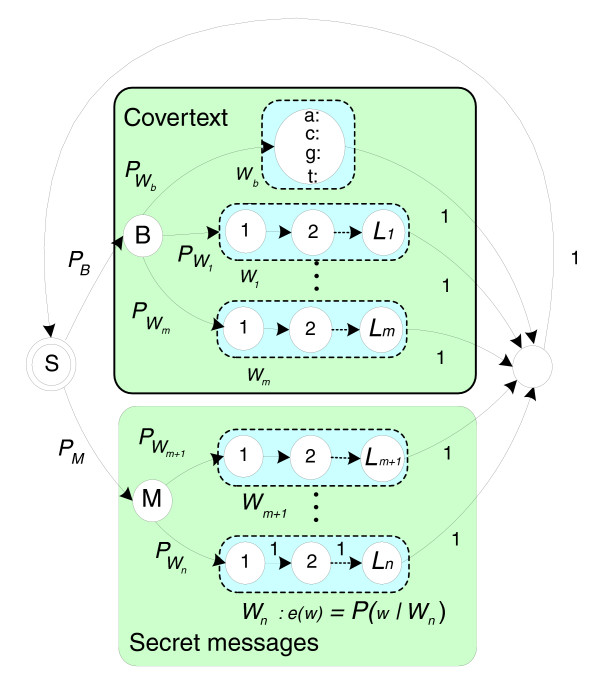
A hidden Markov model for deciphering stegoscripts. It consists of two submodels, the 'secret message model' is for motifs and the 'covertext model' for background words. The blue boxes with dashed outlines each represent a word node, which is a combination of several position nodes. Node *W*_*b *_is a single-base node and always belongs to the covertext model. States *S*, *B*, and *M *do not emit any letter.

To accurately capture the transcriptional mechanism encoded in the regulatory regions requires a complicated grammar, which may be computationally not feasible. To reduce computational complexity, we consider that motifs are used independently. Therefore, we can use a stochastic regular grammar [18], which is equivalent to a hidden Markov model (HMM) [14]. Figure 1 illustrates the model. Beginning with a start symbol, a motif symbol *M *is produced with probability *P*_*M*_, or a background symbol *B *is generated with probability *P*_*B*_. From *M*, a degenerate motif *W*_*i *_is produced, with probability , from the motif subdictionary, and an exact word *w *is generated with probability *P*(*w*|*W*_*i*_). The process for generating a background word from symbol *B *is similar. The generated word is then appended to the script that has been created so far and the process repeats until the whole script is created.

We formally write the model as *G *= {Ψ, Θ, *I*}, where Ψ = {*P*_*B*_,*P*_*M*_,} is the set of transition probabilities, Θ = {Θ_*b*_, Θ_1_, Θ_2_,..., Θ_*n*_} is a set of emission probabilities corresponding to the motifs and words in a dictionary *D *= {*W*_*b*_,*W*_1_,*W*_2_,...,*W*_*n*_}, and *I *= {|*W*_*i *_∈ *D*} is a set of indicators, where



*W*_*b *_is the only word in the model that has a single base. As we never consider a word of single base as a functional element, *W*_*b *_is always a background word, that is,  is always set to 0.

### The WordSpy algorithm

The central problem of deciphering a stegoscript is learning a statistical model with which a stegoscript was created. Assume that a stegoscript *S *was generated from an unknown model 〈D*, G*〉 of a dictionary D* and a grammar G*. With no prior knowledge of the true model, the maximum likelihood estimate, arg max_〈*D'*, *G'*〉 _*P*(*S*|〈*D'*, *G'*〉), is a good approximation of 〈D*, G*〉. However, it is difficult to directly search for arg max_〈*D'*, *G'*〉 _*P*(*S*|〈*D'*, *G'*〉), as a large number of words need to be discovered and many unknown parameters to be optimized. Therefore, we separate the learning process into two phases, 'word sampling' and 'model optimization', and adopt an incremental learning strategy to progressively capture short to long words and gradually build such a model (see Materials and methods).

**Figure 2:**
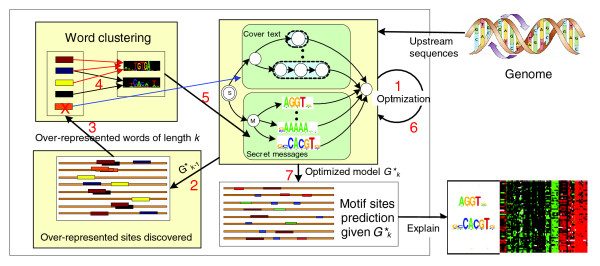
Components and flow diagram of WordSpy. Starting with *k *= 1 and a grammar *G*_0 _with a single word node *W*_*b *_in background, the algorithm goes through the following steps, represented by the red numbers on the figure. 1. Model *G*_*k*-1 _is optimized to  which contains over-represented motifs shorter than *k*. 2. Use  as a base model to detect over-represented exact words of length *k*. 3. Choose over-represented words for word clustering. 4. Evaluate all the words. Select and add background words to the background model. On the basis of similarity, cluster the rest of the words to form degenerate preliminary motifs. 5. Add the preliminary motifs to the motif sub-dictionary and create a new grammar *G*_*k*_. 6. Optimize *G*_*k*_. 7. Apply optimized  to decipher the script and locate motifs.

The procedure for learning the model and subsequently deciphering the regulatory sequences is shown in Figure 2. The overall algorithm starts with the simplest model 〈*D*_1_, *G*_1_〉 with only a background word *W*_*b *_in *D*_1_. At the *k*th iteration, the algorithm first runs word sampling to identify all over-represented words of length *k*. In this process, the algorithm scans the script *S *once to tabulate all the words of length *k *in *S *and their occurrences using a hash table. Every word in the table is then tested against the current best model  which contains over-represented motifs shorter than *k*. A word is considered over-represented if it occurs in *S *more often than expected by . Furthermore, the newly discovered words will be examined (to separate background words) and clustered, if necessary, to form degenerate preliminary motifs. All new words and motifs will be merged with the current best dictionary  to form the next dictionary *D*_*k*_. The model is retrofitted to accommodate the new words, leading to the next grammar, *G*_*k*_. The new grammar *G*_*k *_is then optimized to fit the script. The word statistics are recalculated in the model optimization step and the insignificant words are discarded. The process repeats until the model covers words up to a predefined maximum length.

The classification of real motifs and background words is important to the accuracy of the model. When no extra information is available, we resort to a word significant threshold to select putative motif words. We use the *Z*-score to quantify the over-representation of a word (see 'Word sampling' section in Materials and methods). If more information is available, such as gene-expression coherence in *G*-score and target gene specificity in *Z*_*g*_-score (see 'Motif evaluation' section in Materials and methods), more accurate classification can be made.

### Deciphering an English stegoscript

We evaluated the performance of WordSpy with a stegoscript of English text that contains the first ten chapters (approximately 112,000 letters) of the novel *Moby Dick *embedded within randomly generated covertext (approximately 156,000 letters). This stegoscript was created by Bussemaker *et al*. [15]. We ran WordSpy with different *Z*-score thresholds to find words up to length 15. WordSpy reached its best performance with *Z*-score threshold 6. With covertext removed, the deciphered text contains 16,522 words. Among the total 18,930 words that appear at least twice in the original text, 13,435 (70.9%) words are 100% matched to their corresponding deciphered words, and 15,529 (82%) words overlap at least 50% with their corresponding deciphered words. Only 761 (4.6%) deciphered words match less than 50% to their counterparts in the original text. This result shows that WordSpy can accurately decipher the stegoscript and recover *Moby Dick *from the covertext with high specificity and sensitivity (see Additional data file 1 for a detailed analysis and more results).

### Identifying yeast cell-cycle regulatory motifs

To evaluate the performance of WordSpy on biological sequences, we applied it to discover *cis*-regulatory elements of cell-cycle related genes of *S. cerevisiae *[19]. To avoid bias, we first removed homolog genes using WU-BLAST with an E-value threshold of 10^-12^, resulted in 645 genes in the final set. The promoter sequences were retrieved using the RSA tools [20]. We compared WordSpy with three other methods, MobyDick [15], RSA-tools [21] and Weeder [22], which can handle a large number of sequences. We tuned these programs to get their best possible parameters. The *Z*-score threshold for WordSpy was set to 3. The whole-genome analysis on the specificity of the motifs, *Z*_*g*_-scores, was performed with the promoters of all the genes in *S. cerevisiae*. We also used the yeast gene expression data collected in [23] to calculate the *G*-score for each motif. As shown in Table 1, all known cell-cycle-related *cis*-elements were identified with high ranking in either *Z*_*g*_-score or *G*-score. In contrast, MobyDick failed to discover three of them, and RSA-tools and Weeder missed four of them.

MBF and SBF are predominant TFs in the G1/S phase of the yeast cell-cycle. Their binding motifs, MCB (ACGCGT) and SCB (CRCGAAA) [24], are consistent with the top motifs discovered by WordSpy. Among 199 discovered motifs of length 7, AACGCGT ranks the first in both *Z*_*g*_-score and *G*-score, CGCGAAA is the second in *G*-score and the third in *Z*_*g*_-score, and CACGAAA ranks the 10th in *Z*_*g*_-score and the 17th in *G*-score. Another prominent motif GTAAACA (the 8th in *Z*_*g*_-score and the 10th in *G*-score) has been reported to be the binding motif of Fkh2 (or Fkh1) [25], which is involved in cell-cycle control during pseudohyphal growth and in silencing of MHRa [26]. WordSpy also identifies the binding motifs of Ace2/Swi5 and Met4/Met28 with high *G*-score ranking, and the binding motifs of Mcm1 and Ste12 with high *Z*_*g*_-score ranking.

**Figure 3:**
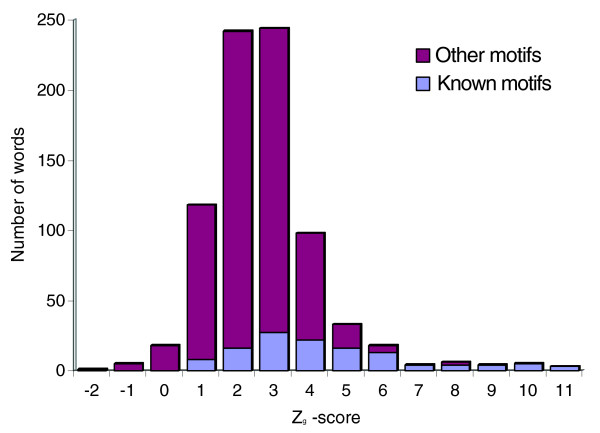
Distribution of discovered yeast motifs of length 8. The *x*-axis is the genome *Z*-score (*Z*_*g*_-score) of a motif, which measures the motif's specificity to the cell-cycle genes. Motifs resembling known ones are marked in blue.

Figure 3 displays the distribution of all discovered motifs of length 8 in reference to the *Z*_*g*_-score. The motifs that overlap with some known motifs by at least six nucleotides are displayed in a different color. This result shows that most of the top-ranking motifs based on the *Z*_*g*_-score resemble known motifs. To facilitate motif selection, we clustered similar motifs. The motifs were first sorted by *Z*_*g*_-score or *G*-score. From the highest to the lowest rankings, we took a motif that had not been clustered as a seed, and grouped it with all the motifs that shared a common substring of length 6 (out of 8 base pairs) with the seed or its reverse complementary. Combining the top 20 clusters of all motifs of length 8 based on *Z*_*g*_-score and *G*-score, all the known motifs are identified (see Tables 3 and 4 in Additional data file 1). All these encouraging results suggest that by combining *Z*_*g*_-score and *G*-score analysis, WordSpy can comprehensively identify real motifs from a large set of regulatory sequences with a high specificity.

### Identifying *Arabidopsis *cell-cycle regulatory motifs

Cell-cycle regulation in plants is more complicated than that in yeast or even mammals. One possible explanation is that the sessile life-style of plants requires a more sophisticated mechanism for growth or development to adapt to adverse environmental conditions [27]. What makes the study of the cell-cycle in plants more appealing is that some plant cells have surprisingly long life spans and are extremely resistant to cancerous conditions. Understanding how plant cells are controlled during development may shed light on the control of human cell proliferation [27].

In this study, we applied WordSpy to identify regulatory elements of 1,081 cell-cycle regulated genes of *A. thaliana*, which were identified by a high-throughput expression profiling experiment [28]. After having removed homologous genes with an E-value threshold of 10^-12^, we had 1,030 genes left for analysis. The promoter sequences were obtained from TAIR database [29]. We ran WordSpy to find motifs with lengths up to 10. The *Arabidopsis *whole-genome transcription-profiling data under normal growth conditions from the Weigel lab [30] were used to calculate motif *G*-scores.

**Figure 4:**
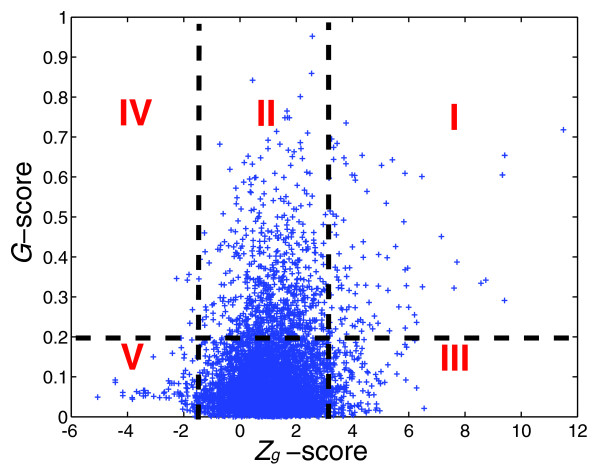
Distribution of all discovered motifs from *Arabidopsis *cell-cycle-related genes. The *x*-axis is the genome *Z*-score (*Z*_*g*_-score) of a motif, which measures the motif's specificity to the cell-cycle genes. The *y*-axis is the *G*-score of a motif, which measures the coherency of the expression profiles of the genes whose promoters contain the motif.

Figure 4 shows the distribution of 5,277 discovered over-represented words over gene specificity in *Z*_*g*_-score (*x*-axis) and gene expression coherence in *G*-score (*y*-axis). We considered words with a *G*-score greater than 0.2 as biologically significant, and used *Z*_*g*_-score thresholds of greater than 3.0 or less than -1.0 to select cell-cycle-related or unrelated motifs. With these criteria, motifs are split into six categories, as shown in Figure 4. The motifs in region I are putative cell-cycle-related motifs that we are mostly interested in. Region II also contains many putative binding motifs for cell-cycle genes, which may not be specific to cell-cycle processes. The motifs in region IV are putative motifs that are more plentiful in non cell-cycle genes. The motifs in regions III and V are the ones that are statistically significant although their target genes do not express coherently. We can consider the rest of the words in the middle region as background words as they do not satisfy either criterion.

**Figure 5:**
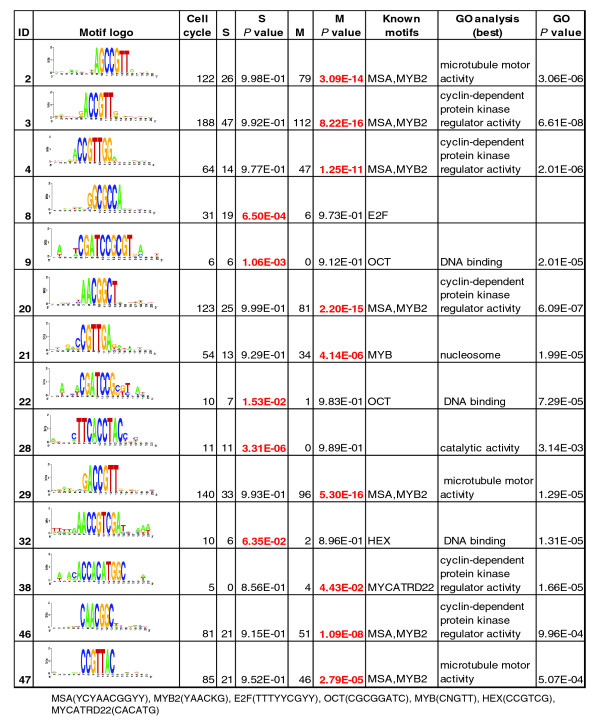
Selected putative *Arabidopsis *cell-cycle-related motifs. ID, the ranking of a motif in the overall list. The third column gives the number of cell-cycle genes whose promoters contain the motif. The following four columns are the number of target genes in S and M phases of the cell cycle and the corresponding *P* value. GO analysis gives the functional group with the best *P* value, which is shown in the last column.

There are 110 motifs in region I of Figure 4 (see Tables 5 and 6 in Additional data file 1). We clustered them to obtain 55 motifs (see Additional data file 2). We selected 14 of the 55 motifs, which are similar to some known motifs listed in the plant motif databases PLACE [31] and PLANTCARE [32], and present them in Figure 5.

To further evaluate whether WordSpy can indeed find functional *cis*-regulatory elements, we analyzed these 55 clustered motifs with respect to different cell-cycle phases. The expressions of 247, 343, 131, and 247 of the 1,081 cell-cycle genes peak in G1, S, G2, and M phases, respectively [28]. On the basis of this target gene distribution in each phase, we calculated the specificity of each motif to every phase of the cell cycle. For example, 79 of 122 target genes containing motif 2 (ID = 2, Figure 5) are M-phase genes. When randomly selecting 122 genes from the set of cell-cycle genes, the chance to have 79 M phase genes is less than 3 × 10^-14^. Therefore, motif 2 is very likely to be an M-phase motif. Surprisingly, all the motifs in Figure 5 have very low *p *values in either M phase or S phase. More interestingly, most motifs with low *p *values in M phase match well with the mitotic-specific activation (MSA) elements (consensus YCYAACGGYY) [33], and the motifs with low *p *values in S phase resemble motifs E2F (TTTYYCGYY) [34], Octamer and Hexamer [35], which are known S-phase motifs.

Furthermore, to reveal possible functions for each of the 55 motifs, we calculated the enrichment of gene ontology (GO) terms [36] within the genes containing the motif (see Materials and methods). Figure 5 shows that almost every motif has some enriched functional categories (*p *value < 1e-2). The most common functional category is the cyclin-dependent protein kinase regulator activity (CDK). Interestingly, many motifs related to CDK are MSA elements or resemble MYB-like motifs, suggesting that MYB-like TFs regulate cyclin kinase-like proteins in G2M phase of the cell cycle. Motif 28 (TTCACCTAC, Figure 5) does not match with any known motif. However, all its 11 target genes peak in S phase, and all seven target genes with GO annotations are related to catalytic activity, implying that this is a novel functional motif. We report all new putative functional motifs in Additional data file 2.

#### MSA motifs are position dependent

**Figure 6:**
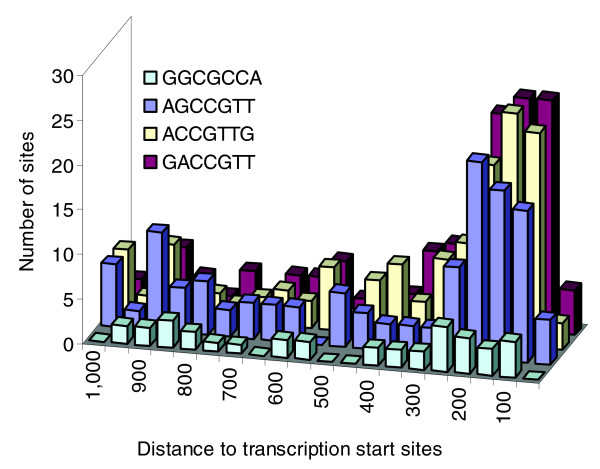
Distribution of the locations of putative *Arabidopsis *motifs. The location distribution of the top four putative motifs of length 7 in the promoters of *Arabidopsis *cell-cycle genes is shown.

The top four motifs of length 7 ordered by *G*-score - AGCCGTT, GACCGTT, ACCGTGG, and GGCGCCA - have both significant *Z*_*g*_-score (> 3.0) and *G*-score (> 0.2). The first three of these motifs resemble MSA elements (consensus CYAACGGYY) [33]. We investigated their position distribution on the promoters of the cell-cycle genes containing the motifs. The result is shown in Figure 6. Three MSA motifs - AGCCGTT, GACCGTT and ACCGTTG - are significantly over-represented near the transcription start sites (TSSs).

**Figure 7:**
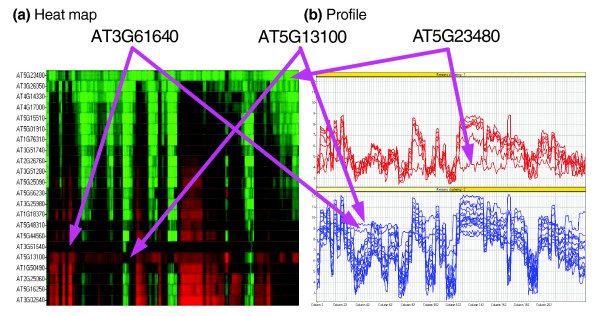
Expression patterns of *Arabidopsis *genes associated with ACTAGCCGTT. The gene-expression profiles are highly coherent except three outliers - AT3G61640, AT5G13100, and AT5G23480. **(a)** Heat-map analysis of microarray expression patterns. **(b)** Profile analysis of microarray expression patterns. Expression profiles are clustered into two groups. The profiles in both red and blue have similar patterns, but the profiles in red have relatively low values.

**Figure 8:**
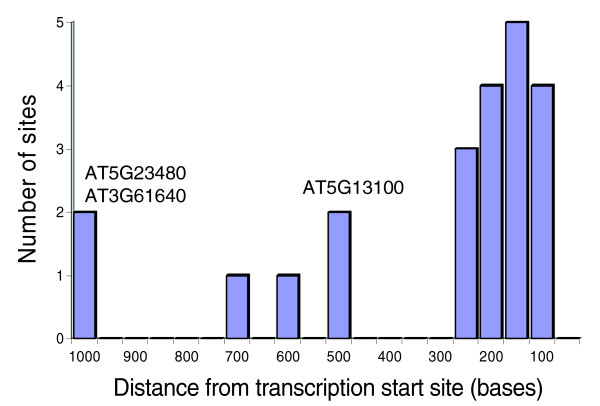
Distribution of the positions of the motif ACTAGCCGTT in the promoters of *Arabidopsis *cell-cycle genes.

We further studied the most significant motif of length 10, ACTAGCCGTT, which is ranked the first in *Z*_*g*_-score (11.4) and the second in *G*-score (0.718) (see Table 5 in Additional data file 1). Figure 7 shows the expression patterns of the genes whose promoters contain ACTAGCCGTT on either strand. Both heat-map and profile chart demonstrate a highly coherent expression pattern, except for three outliers, AT3G61640, AT5G13100, and AT5G23480. Remarkably, the loci of the motif on these outliers are far away from their TSSs, as shown in Figure 8. Moreover, these cell-cycle genes, except the outliers, are all M-phase related according to the experiment in [28]. These results suggest that MSA motifs are position dependent, and usually close to TSSs.

#### E2F binding motifs may vary in cell-cycle related and unrelated genes

Various studies have shown that in addition to the cell cycle, the genes containing binding motif E2F appear in many functional categories including transcription, stress defense, and signaling [37]. As expected, we also identified many E2F-like motifs in region II. Table 2 shows the discovered motifs that match to the known E2F binding elements (consensus TTTYYCGYY) [34]. The motifs in cluster 1 are in the motif region I of Figure 4 with *Z*_*g*_-score greater than 3.0. This cluster of motifs corresponds to motif 8 in Figure 5. The motifs in cluster 2 are in the motif region II with *Z*_*g*_-score less than 3.0. Obviously, the motifs in cluster 1 are more specific to cell cycle than those in cluster 2. These two sets of motifs differ only by two nucleotides in their core sequences. The motifs that are more cell-cycle specific have 'GG' in the middle (TTT**GG**CGCC), whereas the motifs that are abundant in the genome contain 'CC' in their core sequences (TTT**CC**CGCC). Among the cell-cycle genes, TTT**GG**CGCC appears in 14 promoters and TTT**CC**CGCC in 10 promoters. In the whole genome, 100 genes have TTT**GG**CGCC in their promoters and 257 genes have TTT**CC**CGCC.

In summary, these observations indicate that the preferential cell-cycle-related E2F motif is TTT**GG**CGCC, and the non-cell-cycle related E2F motif is TTT**CC**CGCC. In other words, the E2F binding motifs differ based on whether or not they are cell-cycle related. Our results also demonstrate that the WordSpy method can detect such subtle and important difference in regulatory elements.

### Finding discriminative motifs

Given two sets of scripts or sequences, a discriminative motif is such a motif that is over-represented in one script but not in the other. WordSpy is, in essence, an algorithm for finding discriminative motifs, because of its intrinsic feature of modeling motifs and background words in an integral model. Here, background words can be extracted from one set of sequences (negative set), while the discriminative motifs are identified from another set of sequences (positive set).

We applied WordSpy as a discriminative algorithm to find regulatory motifs in *S. cerevisiae*. We constructed positive and negative sequence sets based on the ChIP-chip experiments of Lee *et al*. [38]. For a particular TF, we selected as the positive dataset those promoters that the TF could bind to with *p *values < 0.01 in the ChIP-chip experiments and as the negative dataset those promoters with *p *values > 0.99. We also applied two widely used algorithms, MEME [5] and AlignACE [7] to the same data. MEME was executed with a sixth-order Markov model on the yeast noncoding regions as background. Table 3 lists the motifs that are closest to the known cell-cycle-related motifs from these three algorithms. As shown, WordSpy not only found all known motifs for each TF but also the known motifs of cofactors. MEME and AlignACE were able to find most known motifs, but missed some binding sites of cofactors.

### Evaluation with a benchmark study

Recently, Tompa *et al*. [17] developed a benchmark of a set of well-curated regulatory sequences and *cis*-regulatory elements of budding yeast, fruit fly, mouse, and human for evaluating motif-finding algorithms. They introduced seven statistical measurements to assess the performance of 13 motif-finding programs. An interesting observation on their results is that the enumeration-based methods, represented by Weeder [22] and YMF [8], outperformed the model-based approaches, represented by MEME [5] and AlignACE [7].

Almost all the sets of sequences in the benchmark are relatively small; none of them has more than 35 sequences. Aimed at finding motifs from a large number of sequences, for example, more than 1,000 promoters of genes related to cell cycles in *Arabidopsis*, WordSpy was not originally designed to deal with a small number of sequences. Nevertheless, it can be used to find motifs from a small set of sequences and has a very competitive performance, as we show here. We applied WordSpy to the sets of sequences in the benchmark and compared it with the other programs studied by Tompa *et al*. [17]. For fair comparison, we did not use gene-expression information in WordSpy, but rather used only genomic sequences to calculate the *Z*_*g*_-scores. Moreover, although WordSpy discovered a set of motifs for each sequence set, we reported the most significant motif with some selection criteria. For all the experiments, we built a dictionary up to word length 10. Then we filtered out the motifs with *Z*_*g*_-scores less than 4. Finally, we selected the motif with the highest *Z*-score or *Z*_*g *_-score depending on their site distributions. We always chose the ones that are close to the TSSs.

**Figure 9:**
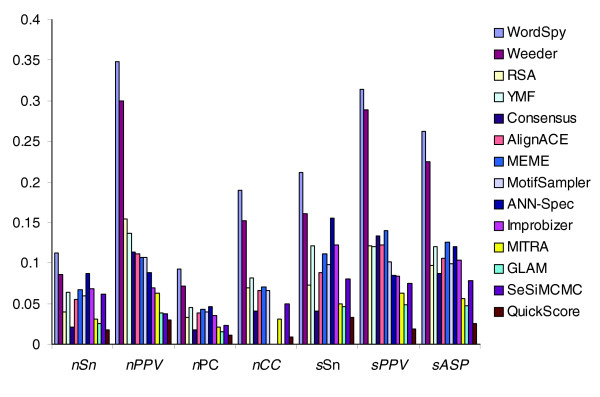
The results of a comparison of 14 motif-detection programs on a benchmark study [17]. At the nucleotide level, sensitivity (*nSn*), positive predictive value (*nPPV*), performance coefficient (*nPC*), and correlation coefficient (*nCC*) were measured. With *nTP*, *nFN*, *nFP *and *nTN *as nucleotide-level true positive, false negative, false positive, and true negative, respectively, *nSn *= *nTP*/(*nTP *+ *nFN*); *nPPV *= *nTP*/(*nTP *+ *nFP*); *nPC *= *nTP*/(*nTP *+ *nFN *+ *nFP*); and *nCC *= (*nTP*·*nTN *- *nFN*·*nFP*)/. At the site level, sensitivity (*sSn*), positive predictive value (*sPPV*), and average site performance (*sASP*) were measured. With *sTP*, *sFN*, *sFP *as site-level true positive, false negative, and false positive, respectively, *sSn *= *sTP*/(*sTP *+ *sFN*); *sPPV*) = *sTP*/(*sTP *+ *sFP*; and *sASP *= (*sSn *+ *sPPV*)/2.

**Figure 10:**
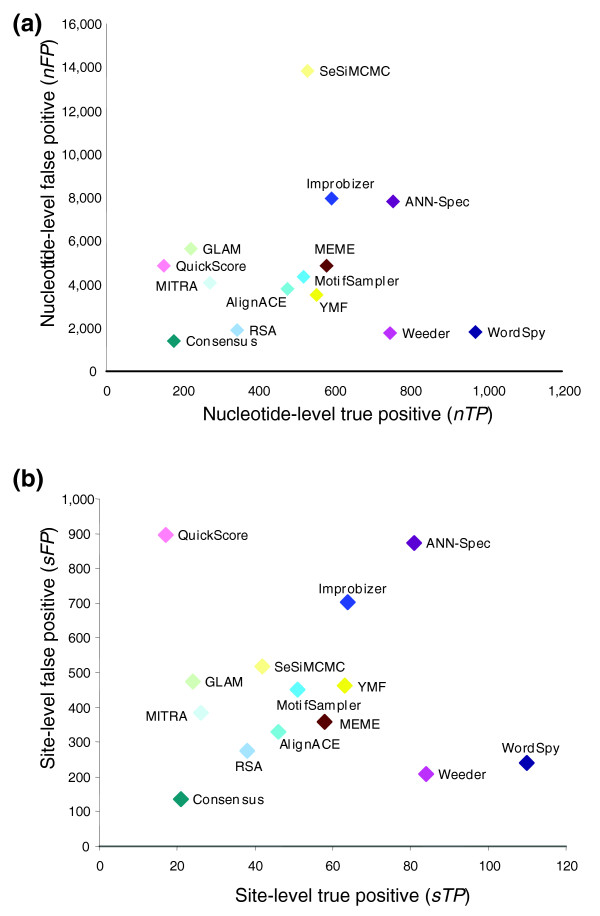
True positives and false positives of the 14 motif-detection programs compared. **(a)** Nucleotide-level true positive (*nTP*) is the number of nucleotide positions in both known sites and predicted sites; nucleotide-level false positive (*nFP*) is the number of nucleotide positions not in known sites but in predicted sites. **(b)** Site-level true positive (*sTP*) is the number of known sites overlapped by predicted sites; site-level false positive (*sFP*) is the number of predicted sites not overlapped by known sites.

Figure 9 shows the comparison results of WordSpy with the 13 programs (Weeder [22], YMF [8], RSA-tool [21], QuickScore [39], AlignACE [7], ANN-Spec [40], MEME [5], Consensus [6], MIRTA [41], GLAM [42], Improbizer [43], MotifSampler [44], SeSiMCMC [45]) on the seven statistics introduced in [17]. A detailed description of these statistics is available on the benchmark website [46]. As shown in Figure 9 and Additional data file 3, WordSpy outperforms the other programs by all the measures. Figure 10 shows true positive versus false positive in both nucleotide level and site level for all the programs. WordSpy has the highest numbers of true positives and relatively low numbers of false positives in both cases. The success of WordSpy may be due to the following reasons. First, WordSpy aims to discover all over-represented motifs; the chance of it missing a significant motif is low. Second, the *Z*_*g*_-scores computed in WordSpy help it to select the right motifs that are specific to a given set of sequences. Third, WordSpy uses a strategy of first searching for over-represented exact words and then combining them to form degenerate motifs. This strategy makes the motif representation in WordSpy more stringent than that in the other methods, and as a result, it has a smaller false-positive rate. Note that WordSpy performs better on the budding yeast and human datasets than on the fruit fly datasets.

## Conclusion

We propose a new approach to the challenging problem of genome-wide motif finding, which combines a novel steganalysis method for discovering over-represented motifs and methods for selecting biologically significant motifs. By taking a steganalysis perspective on the motif-finding problem, we were able to accurately identify a large number of motifs of nearly optimal lengths. By considering all the genes of interest altogether, we avoided the problem of subjectively partitioning the genes into small clusters, which may make some motifs difficult to detect. By applying our approach to all cell-cycle-related genes in budding yeast and *A. thaliana*, we demonstrated its power as an effective genome-wide motif finding approach that compared favorably to many existing methods.

The core motif-finding algorithm, WordSpy, combines both word counting and statistical modeling. Like word-counting methods, WordSpy can simultaneously detect a large number of putative motifs. Unlike the existing word-counting methods, however, the wording-counting procedure of WordSpy is progressive and retrospective. It considers short to long words, adjusts the over-representation of shorter words after examining longer ones, and subsequently eliminates not truly over-represented shorter words. As a result, WordSpy produces fewer spurious motifs and is able to find motifs with optimal lengths. Furthermore, instead of using statistical models to characterize a small number of motifs with multiple local alignments, WordSpy models a large number of motifs, their compositions, and their usage to fit to the whole of the given sequences. Consequently, all significant words in regulatory regions can be identified.

WordSpy is a dictionary-based approach, which was initiated in the innovative MobyDick algorithm by Bussemaker *et al*. [15]. Nevertheless, we significantly extended their work in many important aspects. First, we took a novel steganographic view of the problem of motif finding. This allows us to combine a grammar with a dictionary in a statistical model to capture both conserved motifs and background words. Second, WordSpy accurately quantifies the over-representation of a word by considering the probability that the word can be generated by the best model that has been built so far, whereas MobyDick computes the over-representation by counting the occurrences of a word in a large synthetic dataset. Third, WordSpy considers only those words that occur in the given sequences without enumerating all possible words, which saves a substantial amount of computation, especially for long words.

In the current implementation of WordSpy, we assumed that the motifs and words in a dictionary were used independently. For some applications, however, spatial relationship among motifs may be biologically important. For such cases, we may resort to a more complex grammar, such as stochastic context-free or context-sensitive grammar [18]. However, the incurred computational cost could be prohibitively high for even small problems. A more efficient way to capture motif correlations is to construct motif modules using the motifs identified by a simple grammar model. Similar post-processing strategies have been proposed [47,48].

In this research, we adopted two schemes to measure the biological significance of motifs. One is the expression coherence of the genes whose promoters contain a motif, and the other is the specificity of a motif to the genes of interest with respect to the rest of the genome. Similar ideas have been proposed [49]. As shown in this study, these two biological relevance measures are effective in identifying cell-cycle-related TF-binding motifs of yeast and *A. thaliana*. However, we need to caution that a high *G*-score may not necessarily and sufficiently mean a good motif, a similar restriction to the clustering-first approaches, and that gene-expression information may not be available for all genomes. Therefore, we suggest using the *Z*_*g*_-score as the major criterion, and the *G*-score and other information as supports.

In this study, we applied our approach to identify significant *cis*-elements from sequences of a single species. Like most algorithms that use information of a single species, WordSpy may be vulnerable to noisy promoter sequences as a result of the uncertainty of the annotation, especially in the genomes of higher eukaryotes. A comparative approach may have an advantage in such situations by utilizing conservation information from multiple species. Therefore, we will consider using evolutionary information to improve our method in future work. Nevertheless, computational tools for large-scale *de novo *motif finding for a single species are still important, especially for applications where no sequences of closely related species are available and for problems where species-specific motifs are needed. It is interesting to note that single-species motif finding can be competitive when compared with comparative genomics methods using multiple species [50].

## Materials and methods

### Word sampling

The goal of word sampling is to discover over-represented motifs as completely and accurately as possible. Word sampling determines the structure of the model and initializes its parameters. For biological sequences, a regulatory motif is usually represented by a series of position profiles, each of which is the distribution of four nucleotides at that position. In our model, the emission probability of each position node is equivalent to such a profile. However, such motifs, named as 'profile motifs', exist in a continuous space. It is almost impossible to comprehensively search for all over-represented profile motifs directly. Here, we combine methods of word counting and statistical modeling. We apply a word-counting method to detect over-represented words in the discrete sequence space of four nucleotides, and then cluster similar words to form a profile motif. All resulting profile motifs will be further improved in the model optimization phase.

We develop an efficient algorithm for word sampling to identify all over-represented words of length *k *in the sequence space against the optimal model  in linear time and linear space complexity. The algorithm scans the script *S *once, tabulates, using a hashing scheme, all exact words of length *k *in *S*, and computes their over-representativeness. A word is considered over-represented if it occurs more frequently in *S *than it could be generated by the current best model . We measure the over-representativeness by a *Z*-score. Let *N*_*w *_be the number of occurrences of a word *w *in *S *and random variable _*w *_be the number of occurrences of *w *in a script with the same length as *S *which were supposedly generated by model . Denote *E*(_*w*_) and *σ*(_*w*_) as the mean and standard deviation of _*w*_. The *Z*-score of *w *is defined as *Z*_*w *_= (*N*_*w *_- *E*())/*σ*(_*w*_). It is nontrivial to compute the statistics of random variable _*w*_. Consider a word *w *of length *k *in a sequence of length *L *generated by model . There are various ways to produce *w *using the model, for example, by concatenating words of a single letter, or by merging a word's suffix with another word's prefix. To compute the expected number of occurrences of *w*, *E*(_*w*_), we define (*i*) (and respectively (*j*)) to be the set of words in  whose suffixes (and respectively prefixes) match the first *i *(and respectively the last *j*) letters of *w*. The expectation *E*(_*w*_) can be computed as



where



and *w*_[*j*,*k*] _represents the subsequence of *w *from its *j*th to *k*th positions,  is the transition probability of motif *W*_*u*_, and  and  are the emission probabilities of the last *i *and first *j *positions of Θ_*u*_, respectively. The computation of *σ*(_*w*_) is complex and costly [51,52]. Following the practice in the existing methods, in our current implementation, we approximate *σ*(_*w*_) by *E*(_*w*_).

All the words with Z-scores greater than a threshold are considered over-represented. Thereafter, all new words are classified into background words or motif words with some motif evaluation methods. Two evaluation methods will be described in the section on 'Motif evaluation' below. After evaluation, background words are added to background sub-dictionary of . The motif words are further clustered to form profile motifs.

The current implementation of word clustering is a greedy algorithm. Let *C *= {*w*_1_,*w*_2_,...,*w*_*m*_} be a set of words of length *k*, sorted in a non-increasing order of their *Z*-scores. From the beginning to the end of list *C*, we take a word *w*_*j *_as a seed and search the words in *C *that match *w*_*j *_by at least *κ *letters, where *κ *is determined so that the chance of two random words of length *k *having *κ *matched letters is less than 0.001. All such matched words are then merged with *w*_*j *_and subsequently removed from the seed candidate list. The procedure terminates after all seeds have been examined. This heuristic assumes that the degeneracy is uniform over all positions of a motif. Regulatory motifs may, however, have one or two core parts that are more conserved than their flanking sequences, which sometimes may be 'do-not-care' positions. Fortunately, the current model  keeps all short but over-represented motifs that may include those possible cores of longer motifs. We can also make a nonuniform seed by parsing a word in *C *through , finding some cores (substrings), fixing the seed at those core positions, and allowing mismatches at the other positions. Note that these word clusters are not final. During the model optimization, word clusters are dynamically changed as profile motifs are updated.

At the end of word sampling, the new profile motifs are added to the motif sub-dictionary of  (*I*_*W *_is set to 1) to form the next dictionary *D*_*k*_. The model is retrofitted to accommodate the new motifs, leading to the next grammar *G*_*k*_. The new model *G*_*k *_is then optimized in the model optimization phase. The overall process repeats until the model covers motifs up to the maximum length.

### Model optimization

The goal of model optimization is to optimize the profile motifs as well as their usage probabilities. In this phase, motif statistics are recomputed and insignificant motifs are discarded. Given a stegoscript *S *and a grammar *G*_*k *_= (Ψ, Θ, *I*), where *I *has been determined in word sampling, an optimized grammar  can be derived using the expectation maximization (EM) algorithm [53].

Without loss of generality, we view a set of sequences as a long sequence *S *= *s*_1_*s*_2_...*s*_*q*_. Let  = (Ψ^(*t*)^, Θ^(*t*)^) be *G*_*k*_'s parameters in the *t*th iteration. We can adopt a dynamic programming forward-backward algorithm [14] to compute the most probable state when observing *s*_*l *_∈ *S*. Specifically, we compute the probability of observing *s*_*l *_at the *j*th position of a motif *W *given Ψ^(*t*) ^and Θ^(*t*) ^as follows,



where W[*j*] is the *j*th position of *W*, *f*(*μ*) is the probability of observing *S *up to *s*_*μ *_(inclusive) given , *μ *= *i *- *k*, *ρ*_*W *_= *P*_*W*_(*I*_*W*_*P*_*M *_+ (1 - *I*_*W*_)*P*_*B*_), and *b*(*ν *+ 1) is the probability of observing *S *from *S*_*ν *+ 1 _(inclusive) to the end of *S*, *ν *= *i *- *k *+ *l*(*W*), and *l*(*W*) is the length of *W*. Function *f*(*i*) can be recursively computed as *f*(*i*) = ·*τ*_*W*_(*i *- *l*(*W*) + 1, *i*)·*f*(*i *- *l*(*W*)). Similarly *b*(*i*) can be computed as *b*(*i*) = ·*τ*_*W*_(*i*, *i *+ *l*(*W*) - 1)·*b*(*i *+ *l*(*W*)). Evidently, *P*(*S*|) = *f*(*q*) = *b*(1).

With this posterior probability, we can easily have,  and , where  is the average number of *W*_*i *_likely to be observed and (*ς*, *j*) is the average number of letter *ς *likely to be observed at the *j*th position of *W*_*i*_, in all the possible parses of *S *given Ψ^(*t*) ^and Θ^(*t*)^. On the basis of the maximum likelihood principle, a model that fits the data better will have the following parameters,



where Λ is the alphabet, *ς *∈ Λ, *j *= 1,2,...,*l*(*W*_*i*_), *l*(*W*_*i*_) is the length of *W*_*i*_, and *δ *(*x*, *y*) equals 1 if *x *= *y*, or 0 otherwise. The model optimization is done iteratively using equations in (3) until convergence.

In this procedure, the computation of the forward-backward algorithm becomes more costly when the number of motifs in the dictionary increases because its time complexity is *O*(*L*·*N*), where *L *is the sequence length and *N *the size of the dictionary. We introduce a hash scheme to index a word *w *directly to the profile motifs that may emit *w *in the dictionary, which reduces the average cost of forward-backward algorithm to *O*(*αL*), where *α *is the average link length of the words in the hash table. The links are initially created during word clustering. When a profile motif is generated from a word cluster, every word in the cluster will add a link to the motif in its hash field. Because a word may appear in multiple clusters, its hash field may contain multiple links. These links will also be dynamically changed at the end of each iteration, as the profile motifs are updated.

### Motif evaluation

WordSpy is designed to identify a complete list of putative motifs and usually gives a large number of significant words. How to separate true motifs from background words is critical. As the covertext consists of random strings, a proper *Z*-score threshold can be used to filter out most background words. However, the regulatory regions of a genome are not purely random. There exist many highly over-represented pseudo-motifs that make it harder to find real, functional motifs. Fortunately, functional motifs often have intrinsic properties that make them separable from spurious ones.

#### Specificity to the target promoters

An extracted motif cannot be considered as a genuine motif specific to the genes of interest if it is prevalent in other promoter regions of the genome. We utilize this property to discriminate real motifs from fake ones. This is done by a whole genome analysis with a Monte Carlo simulation of thousands of runs. In each run, a set of promoters are randomly selected from the genome and the occurrence of a motif is counted. A genome *Z*-score, shortened as *Z*_*g*_-score, is calculated to measure the specificity of the motif to the target promoters from which it was discovered with respect to randomly selected promoters. A high positive *Z*_*g*_-score is desired, as it means that the motif is unlikely to be a background word.

#### Gene-expression coherence

Statistically a set of genes sharing a motif will have more similar expression profiles than a set of arbitrary genes. Therefore, we can measure the likelihood of a motif being biologically meaningful by the coherence of the expressions of all the genes whose promoters contain the motif. We use the average coherence of pairwise gene expression to measure the coherence of a set of expression profiles. We call this measure the *G*-score, where *G *stands for genes. A higher *G*-score indicates a more biologically significant motif. The pairwise gene-expression coherence can be measured in many ways, such as Euclidean distances and Pearson correlation coefficients. Here, we present our results using Pearson correlation coefficients. We have also analyzed the expression coherence score in [49] and a normalized version of the *G*-score. Our results on yeast (see Additional data file 1) indicate that the simple Pearson correlation-coefficient *G*-score works slightly better than the other two.

### GO functional analysis

To determine whether any GO terms are enriched in a specified list of genes, we use GO::TermFinder perl module[54] to calculate a *p *value with accumulative hypergeometric distribution,



where *N *is the total number of genes, *M *is the number of genes annotated to have a specific function, *n *is the number of genes tested, and *k *is the number of genes tested which are annotated to have the specific function. The *p *values are adjusted by Bonferroni corrections for multiple tests [55]. GO annotations of *Arabidopsis *were retrieved from TAIR database (version January 2006) [56]. The significantly enriched functional categories were discovered with a false-discovery rate (FDR) of less than 0.05 [57].

### WordSpy webserver

An online server has been set up for the WordSpy algorithm to support direct access to the software at [58].

## Additional data files

Additional data are available with this article. Additional data file 1 contains supplementary material; Additional data file 2 contains *Arabidopsis *cell-cycle motifs; Additional data file 3 contains evaluation results on the benchmark.

## Supplementary Material

Additional file 1 Click here for file

Additional file 2 Click here for file

Additional file 3 Click here for file

## Figures and Tables

**Table 1:** Identified known motifs in the promoters of 645 yeast cell-cycle genes

Transcription factors	Known motifs	WordSpy	*Z*-score	*Z*_*g*_-score	*G*-score	Rank	MobyDick	RSA	Weeder
Ace2, Swi5	RRCCAGCR [19]	CCAGC(-)	5.4	5.2	0.0363	8/3/29	ACCCGGCTGG	N/A	N/A
		GCCAGC(+)	5.3	2.6	0.0551	36/4/58			
		AGCCAGC(+)	4.6	2.5	0.0688	75/13/199			
		CCAGCAAA(-)	4.3	3.5	0.113	107/51/867			
		CCAGCAAG(-)	3.9	2.9	0.0976	185/67/867			
		GCCAGCAA(-)	3.9	3.4	0.1872	124/12/867			
		AGCCAGCA(+)	5.7	2.7	0.0929	189/73/867			
	ACCAGC [59, 60]	AACCAGCA(+)	3.8	2.6	0.1983	239/8/867			
									
Swi6, Mbp1	ACGCGT [19, 60]	AACGCGT(+)	13.7	11.3	0.1816	1/1/199	AACGCGT	AAACGCGT	ACGCGT
		GACGCGTC(+)	9.3	4.9	0.2106	41/4/867	ACGCGTC	ACGCGTAA	ACGCGTAA
		AAACGCGT(+)	14.6	10.2	0.2093	3/5/867		AACGCGTC	CGACGCGT
		AACGCGTC(*)	10.8	8.9	0.2003	9/7/867		ACGCGTCA	GACGCGTA
		ACGCGTAA(*)	9.6	9.0	0.1341	7/36/867		ACGCGTCG	AAACGCGT
		ACGCGTCA(*)	8.9	7.3	0.1291	15/41/867		AACGCGTT	GACGCGTG
		CAACGCGT(+)	6.3	4.0	0.1014	73/59/867		AACGCGTA	
									
Swi4, Swi6	CACGAAA [19, 60]	CACGAAA(*)	4.6	5.7	0.0623	10/17/199	CGCGAAA	ACGCGAAA	ACGCGAAA
		ACACGAAA(-)	6.6	4.5	0.1081	57/55/867		CGCGAAAA	CACGAAAA
		CACGAAAA(+)	7.1	5.5	0.1053	32/57/867		CACGAAAA	ACACGAAA
	CGCGAAA [60]	CGCGAAA(*)	14.9	10.6	0.132	3/2/199			
		ACGCGAAA(*)	15.2	10.3	0.1733	1/15/867			
		CGCGAAAA(+)	17.7	9.4	0.1352	4/34/867			
									
Fkh1, Fkh2	GTAAACA [25]	GTAAACA(+)	8.2	7.4	0.084	8/10/199	GTAAACA	GTAAACAA	GTAAACAA
		GGTAAACA(+)	7.2	4.6	0.1578	48/21/867		ATAAACAA	AATAAACA
	GTAAACAA [60]	GTAAACAA(*)	9	6.6	0.098	11/66/867		AATAAACA	
	ATAAACAA [60]	ATAAACAA(*)	8.8	5.9	0.0657	23/142/867			
MCM1	TTTCCTAA [25]	TTTCCTAA(+)	5.5	5.2	0.0435	35/307/867	N/A	N/A	N/A
Ste12	TGAAACA [61]	TTGAAACA(*)	4.3	4.2	0.0647	66/145/867	N/A	N/A	N/A
		TGAAACAA(*)	5	4.8	0.0631	46/149/867			
Met4, Met28	TCACGTG [62]	TCACGTG(-)	5	1.7	0.0845	129/9/199	N/A	N/A	N/A
Cbf1		GTCACGTG(-)	5	0.9	0.2205	661/3/867			

The first two columns list the known TFs and the known binding motifs. The next five columns report the results from WordSpy, followed by the last three columns for the results from MobyDick, RSA tools, and Weeder. The motifs discovered by WordSpy are marked with (+) if on the up strand, (-) if on the down strand or (*) if on both strands. Rank is based on *Z*_*g*_-score and *G*-score, where the first number is the ranking on *Z*_*g*_-score and the second is on *G*-score and the third is the total number of discovered motifs of the same length.

**Table 2:** Discovered E2F motifs with *G*-score greater than 0.2

**Motif**	***Z*_*g*_-score**	***Z *-score**	***G *-score**	**Number of occurrences**	**Number of promoters**	**Known motifs**
Word cluster 1:						
TT*GG*CGCCTC(-)	3.768	11.6	0.633	4	4	E2F(TTTYYCGYY)
TTT*GG*CGCCT(-)	4.384	9.5	0.438	5	5	E2F(TTTYYCGYY)
T*GG*CGCC(*)	3.006	5.6	0.255	20	20	E2F(TTTYYCGYY)
						
Word cluster 2:						
TTT*CC*CGCCA(-)	-0.598	12.9	0.508	6	5	E2FANTRNR(TTTCCCGC)
TTT*CC*CGCC(+)	-0.613	4.7	0.289	5	5	E2FANTRNR(TTTCCCGC)
TT*CC*CGC(+)	0.236	5.7	0.285	36	32	E2FANTRNR(TTTCCCGC)
TTT*CC*CGCT(+)	0.227	4.3	0.273	7	7	E2FANTRNR(TTTCCCGC)

Motifs in cluster 1 are in motif region I (Figure 4) with *Z*_*g*_-score greater than 3.0. Motifs in cluster 2 are in motif region II with *Z*_*g*_-score less than 3.0. The motifs are marked with (+) if on the up strand, (-) if on the down strand or (*) if on both strands. Number of occurrences is the number of occurrences of a motif and Number of promoters is the number of promoters containing the motif.

**Table 3:** Discovered motifs using positive and negative data

**Transcription factors**	**Known motifs**			**WordSpy**		**MEME**	**AlignACE**
ACE2	CCAGCA	GCTGG(1)	CCAGC(2)	GCTGGC(1)	AACCAGC(2)	AACCAGCA(7)	AACCAGC(12)
Fkh1	GTAAACA	GTAAACA(1)	TGTTTAC(2)	GTAAACAA(1)	TTGTTTAC(2)	GTAAACAA(1)	AAANGTAAACA(5)
Fkh2	GTAAACA	GTAAACA(1)	TGTTTAC(2)	GTAAACAA(1)	TTGTTTAC(2)	TTGTTTAC(1)	AANRWAAACA(3)
Mbp1	ACGCGT	ACGCGT(1)	AACGCGT(1)	ACGCGTT(2)		AACGCGTT(2)	RACGCGWY(3)
	CRCGAAA	GACGCGA(3)	TCGCGTC(5)	ACGCGAA(6)		n/a	ACGCGWAAAA(9)
Mcm1	TTTCCTAATTAGGAAA	TAGGAAA(1)	TTTCCTAA(9)	TTAGGAAA(10)		CCTAATTAGG(1)	TTNCCNNNTNNGGAAA(1)
Met4	TCACGTG	CACGTGA(1)	TCACGTG(2)			CACGTGA(1)	CACGTGAY(2)
	AAACTGTGG	GTGGC(1)	CCACA(3)	TGTGG(5)	CTGTG(6)	CCACAGTT(3)	AAACTGTGG(4)
		TGTGGC(2)	CCACAGT(3)	GCCACAC(4)	ACTGTGG(5)	AACTGTGG(7)	
Met31	AAACTGTGG	TGTGGC(1)	GCCACA(2)	GCCACAC(2)		TGTGGCG(10)	AAAANTGTGGC(4)
	TCACGTG	CACGTGA(1)	TCACGTG(3)			GCACGTGA(2)	CACGTGANNT(7)
Stb1	ACGCGA	AACGCG(4)	TCGCGTT(3)	TCGCGTT(3)		TTCGCGTT(3)	AACGCSAAAA(3)
	CRCGAAA	TTCGCG(1)	TTTCGCG(1)	TTTGGCG(2)	TTTCGTG(5)	CGCGAAAA(1)	AACGCSAAAA(3)
	ACGCGT	ACGCGT(3)				n/a	n/a
Ste12	TGAAACA	TGAAACA(1)	ATGAAAC(2)	TGAAACAA(2)		TGAAACA(2)	ATGMAAC(13)
Swi4	CGCGAAA	ACGCGAA(1)	GACGCGA(2)	AAACGCG(3)	CACGAAA(7)	GACGCGAA(1)	RACGCGAAAA(2)
	ACGCGT	AACGCGT(10)				n/a	n/a
Swi5	CCAGCA	GCTGG(1)	CCAGC(2)			n/a	n/a
Swi6	ACGCGT	ACGCGT(1)	AACGCGT(2)	ACGCGTT(3)		AACGCGTT(2)	AAACGCGW(4)
	ACGCGA	AAACGCG(5)	CGCGTTT(6)	ACGCGAA(10)	TTCGCGT(12)	TTTCGCG(3)	AAACGCGW(4)

The table lists the motifs found by three algorithms which are closest to the known regulatory motifs of the 12 yeast cell-cycle TFs. Promoters were chosen based on the ChIP-chip experiments of Lee *et al*. [38]. The rankings from each algorithm are included in parentheses. The rankings for WordSpy are among the words of the same length.
