# Supplementary material for: A steganalysis-based approach to comprehensive identification and characterization of functional regulatory elements
Source: Genome Biol. 2006 Jun 20;7(6):R49. doi: 10.1186/gb-2006-7-6-r49 (PMC1779545; doi:10.1186/gb-2006-7-6-r49)
Supplement: Additional file 1 [file gb-2006-7-6-r49-S1.pdf]

Supplemental materials:

Comprehensive Identification and Characterization of Functional Regulatory Elements with a Steganalysis-based Approach

Guandong Wang[1] and Weixiong Zhang[1,2,†]

[1]Department of Computer Science and Engineering

[2]Department of Genetics

Washington University in Saint Louis

Saint Louis, MO 63130-4899, USA

email: gw2@cse.wustl.edu, zhang@cse.wustl.edu

†: Corresponding author: zhang@cse.wustl.edu; phone: (314)935-8788.

Gene Expression Coherence Score (G-score) and test result. Gene expression coherence score (G-score) measures the similarity of a set of gene expression profiles. Co-regulated genes tend to have similar expression profiles over different conditions. We can thus evaluate the likelihood of a motif being biologically meaningful by the coherence of the expression profiles of all the genes whose promoters contain the motif. The expression coherence of a pair of genes can be measured in many ways, such as Euclidean distances and correlation coefficients. However, a good expression coherence of a set of genes is difficult to define. By considering a gene profile as a point in a n dimensional condition space, we have tested three different methods.

The simplest way to define the coherence of a set of genes is to use the average coherence of pairwise gene expression profiles, shorten as ACOP score. In our test, we measured a pairwise gene expression coherence by correlation coefficients. In this case, the G-score measures the average absolute spread of a set of points without considering the number of points in this set.

To take into account of the number of points in the set, we randomly sampled the same number of gene profiles and calculated the sample mean and standard deviation of their ACOP scores. Then a Z-score was calculated to measure the significance of ACOP against the random samples, called SACOP score. SACOP reflects the relative tightness of a set of points.

In the case where a gene cluster is split to two tightly clustered subsets, which are nevertheless remote

from each other, the ACOP or SACOP scores are relatively low. Another approach is to count the number of good pairs as follows. We randomly sampled 100 genes from the entire genome and calculated all pair-wise coherence of their expression profiles. We then defined the fifth percentile of the distribution of these pair-wise coherence as a threshold T. For a set of N genes, we counted the number of good pairs of genes whose expression coherence are above the threshold T. By randomly sampling N genes from the genome and counting the number of good pairs for each sample, we calculated a Z-score, named GP score, to measure how significant the number of good pairs in the original set versus the random samples. This approach can somehow overcome the problem of splitting clusters.

We ran the experiments on the yeast cell-cycle genes with all three methods. The known cell-cycle motifs of yeast and their G-score rankings with different methods are listed in the supplemental table 2. The results suggest that the three methods are comparable. Since the ACOP score is the easiest to compute, we adopt it in our WordSpy implementation.

Deciphering an English stegoscript. We applied WordSpy to a stegoscript ($\sim$268K letters) that had the first ten chapters ($\sim$112K letters) of the novel *Moby Dick* embedded within. Supplemental Fig. 1 shows a small portion of the stegoscript, where the underlined text is the title and first two sentences of Chapter One. We ran WordSpy with different Z-score thresholds and tried to find words of maximum length of 15. We measured performance by the true positive rate (TPR), the percentage of true words discovered over all the words in the original text, and false prediction rate (FPR), the percentage of false predictions in the deciphered text. In measuring these rates, we considered different degrees of matches between a word in the original text and a predicted word in the deciphered text. We defined the *match rate* of a word as the percent of the characters of the word that match to the original word. We considered a prediction correct if its word match rate is greater than a threshold. As summarized in Supplemental Table 1, TPR decreases and FPR increases as the threshold of word match rates increases. Under the most stringent criterion of 100% word match, WordSpy is able to recover $\sim$70% *exact* original words with a false prediction rate of $\sim$19% using Z-score threshold 6. As a comparison, when the word match rate threshold is 50%, TPR increases to $\sim$82% while FPR decreases to $\sim$4.6%.

A close examination showed that the FPR initially decreases and then stays relatively constant as the Z-score threshold increases (Supplemental Fig. 2(a)). When the Z-score threshold is high enough ($>$5.5), most falsely predicted words will be filtered out. On the other hand, the true positive rate (TPR) always decreases as the Z-score threshold increases. The overall best performance seems to be reached around the Z-score threshold of 6 (Supplemental Fig. 2(b)).

To further analyze WordSpy's performance, we tested it on stegoscripts with uniformly random covertext of different sizes. Using the first ten chapters of *Moby Dick*, we generated six scripts with the ratios of covertext to secret messages ranging from 2 to 7. We expect the deciphering problem to become harder as the amount

of covertext increased. Supplemental Fig. 3 shows the results with Z-score threshold 3 on all six scripts. As expected, the true positive rate decreased and the false prediction rate increased as the amount of covertext increases. However, even when the covertext was 7 times as big as the original novel, WordSpy performed reasonably well; it accurately predicted 75% of the original words and had only 53% of its predictions false positive.

To complete our example in Supplemental Fig. 1(a), we show the recovered text in Supplemental Fig. reffig:moby(b), where the identified background words are replaced by dots. Remarkably, the deciphered text is somewhat comprehensible.

Figures and Tables

```
chapterptgpbqdrftezptqtasctmvivwpecjsnis
rmbtqlmlfvetloomingscallmeerishmaelsomey
lqyearstvhnjbagoaxhjtjcokhvneverpmqpmind
howzrbdlzjllonggbhqipreciselysunpvskepfd
jktcgarwtnxybgcvdjfbnohavinglittlezoruno
zsoyapmoneyyvugsgtsqintmyteixpurseiwfmjw
gjnyyveqxwftlamnbxkrsbkyandrnothingcgpar
ticularwtzaoqsjtnmtoqsnwvxfiupinterestzt
imebymonlnshoreggdithoughtyxfxmhqixceojj
zdhwouldsailpcaboutudxsbsnewtpggvjaasxms
vlittleplvcydaowgwlbzizjlnzyxandzolwcudt
hjdosbopxkkfdosxardgcseebbthefzrsskdhma
wateryjikzicimypartmofprtheluworldvtoamf
utitazpisagwewayrqbkioshavebojwphiixofpr
malungipjdrivingpkuyoikrwxoffodhicbnimthe
ixyucpdzacemspleenqbpcrmhwvddyaiwnandad
abkpgzmptoregulatingeetheslcirculationv
```

(a) *Moby Dick* in a random covertext

```
chapter.................................
.............ooming.call...rishmaelsome.
..years....................neverp...mind
how........long.....precisely...........
......................havinglittle......
......money.........in.......purse......
........................and.nothing..par
ticular...........to..........interest.t
ime.......shore...ithought..............
...wouldsailp.about.....................
.little......................and........
.....................seebbthe.........a
water..........part.of..the..world.to...
......pisa...way.......have.........of..
.........driving..........off.......imthe
ix..................pcrmh........nand..
........toregu.ating.ethe..circulati...
```

(b) Deciphered *Moby Dick* from covertext

Figure 1: Deciphering novel *Moby Dick* from a stegoscript. (a) A small portion of the script; the underlined text is the title and first two sentences of Chapter One. (b) Deciphered *Moby Dick*. The identified background words are marked out by dots.

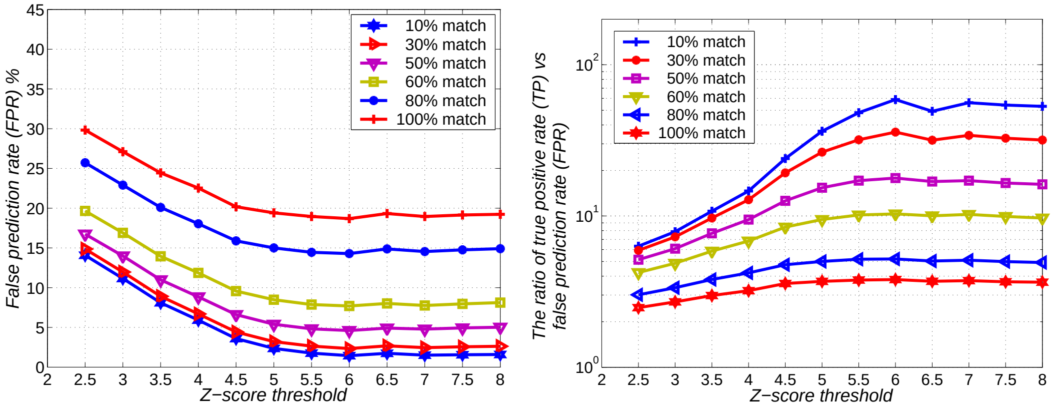

(a) (b)

Figure 2: Evaluation of WordSpy on a stegoscript of *Moby Dick*. (a) False prediction rates on different Z-score thresholds. (b) The ratios of true prediction rate over false prediction rate on different Z-score thresholds. The results are listed for different word matching ratios. 100% match means exactly matching. 50% match means that at least half of a word must be covered by the deciphered text to be considered predicted.

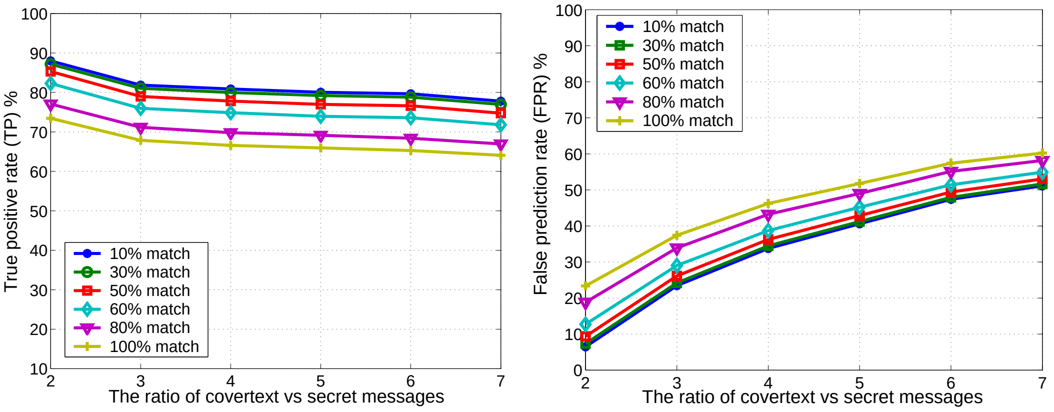

(a) (b)

Figure 3: Prediction results on stegoscripts with different size of covertext. (a) True positive rate on different size of covertext. (b) False prediction rate on different size of covertext.

Table 1: Results on a stegoscript containing the first ten chapters of novel *Moby Dick* for Z-score threshold 6. Total 18930 words are in the original text. Total discovered words in the deciphered text are 16522. *Word match ratio* determines the least percentage of position matches for a true word to be considered correctly predicted. *True words discovered* gives the numbers of true words correctly predicted. *True positive rate* is the percentage of *true words discovered* over the total words in the original text. *False words reported* is the number of words falsely predicted based on different word match ratios. *False prediction rate* is the percentage of false words reported over the total words in the deciphered text.

**Word match ratio (%):**	**10**	**20**	**30**	**40**	**50**	**60**	**70**	**80**	**90**	**100**
**True words discovered:**	16047	16026	15899	15670	15529	15046	14584	14066	13500	13435
**True positive rate (%):**	84.7	84.6	83.9	82.7	82.0	79.4	77.0	74.3	71.3	70.9
**False words reported:**	238	259	387	617	761	1272	1787	2361	3003	3087
**False prediction rate (%):**	1.4	1.5	2.3	3.7	4.6	7.6	10.8	14.2	18.1	18.6

Table 2: Known cell-cycle motifs of yeast and their G-score rankings with different methods.

**Motif**	**ACOP rank**	**SACOP rank**	**GP rank**	**Total # motifs**
TGCTGG	22	60	51	147
GCTGG	10	16	6	30
ACGCGT	1	1	2	147
CACGAAA	47	61	33	419
CGCGAAA	8	4	6	419
ATAAACAA	44	19	43	1015
GTAAACAA	21	12	56	1015
GTAAACA	21	10	72	419
TTTCCTAA	61	77	52	1015
TCACGTG	93	167	142	419
TGAAACA	55	57	162	1015

Table 3: Top clusters of yeast cell-cycle motifs of length 8 based on Z_g-scores. The clusters are ordered as they were created (see text). **#words** is the number of words in a cluster.

	**Representative Motifs**	**#words**	**Known TFs**
1	ACGCGAAA|CGCGAAAA|AACGCGAA	15	Swi4,Swi6
2	AAACGCGT|AACGCGAA|ACGCGTAA|TTACGCGT	21	Swi4,Swi6,Mbp1
3	CGCGACGC|GCGACGCG	6	N/A
4	GTAAACAA|ATAAACAA|AATAAACA	19	Fkh1,Fkh2
5	AAAACAAA|GTAAACAA|ATAAACAA|AAAAACAA	30	Fkh1,Fkh2
6	GAGACGCG|AACGCGTC|GACGCGTT	13	Swi6,Mbp1
7	CACGAAAA|CGCGAAAA|TTTCGTGT	12	Swi4,Swi6
8	TTTCCTAA|AAAGGAAA|CTTTCCTA	9	Mcm1
14	TTTACGCG|ACGCGTAA|TTACGCGT|CACGCGTA	4	Swi6,Mbp1
19	TGTTTCAA|TGAAACAA|TTGAAACA	10	Ste12
33	CCAGCAAA|GCCAGCAA|CTTGCTGG|AGCCAGCA	13	Ace2,Swi5
67	CACGTGCA|CACGTGGG|CCACGTGC|CACGTGAC	8	Met4,Met28,Cbf1

Table 4: Top clusters of yeast cell-cycle motifs of length 8 based on G-scores. The clusters are ordered as they were created (see text). **#words** is the number of words in a cluster.

	**Representative Motifs**	**#words**	**Known TFs**
1	AGACGCGA|GACGCGTC|AACGCGTC	15	Swi4,Swi6,Mbp1
2	TTTTGGCG|TTGGCGTT|ATTTTGGC	13	N/A
3	CACGTGAC|CACGTGGG|TCACGTGC	7	Met4,Met28,Cbf1
4	AAACGCGT|AACGCGTC|AAAACGCG|CAAACGCG	21	Swi6,Mbp1
5	GCGTCGCG|GCGACGCG	6	N/A
6	AACCAGCA|TTTGCTGG|CTTGCTGG|AGCCAGCA	6	Ace2,Swi5
7	TTGCTGGC|AACCAGCA|TTTGCTGG	7	Ace2,Swi5
9	GGTAAAGG|GGTAAACA	6	Fkh1,Fkh2
11	CGCGTGAA|CACGCGTA	2	Swi6,Mbp1
16	TTCGTGTT|TTTCGTGT|CACGAAAA	3	Swi4,Swi6
19	CGCGAAAA|ACGCGAAA|AACGCGAA|TTCGCGTC	18	Swi4,Swi6
31	GTAAACAA|GGTAAACA|ATAAACAA|TGAAACAA	19	Fkh1,Fkh2
49	TTTGAAAC|TGTTTCAA|TGAAACAA	8	Ste12

Table 5: Putative motifs of Arabidopsis cell-cycle genes with Z_g-score greater than 3.0 and G-score greater than 0.2. **#Occ.** and **#Pr.** are the number of occurrences of a motif and the number of promoters containing the motif. *Continued on Table 6.*

**Motif (complimentary)**	Z**-score**	Z_g**-score**	G**-score**	**#Occ.**	**#Pr.**	**Known motifs**
GAATCCGGCG(CGCCGGATTC)	11.8	3.771	0.735	4	4	ARR1AT(NGATT)
ACTAGCCGTT(AACGGCTAGT)	18.9	11.496	0.718	14	13	
GACCGTTGCT(AGCAACGGTC)	12.1	3.258	0.695	6	6	MYBCORE(CNGTTR) MYB2(YAACKG)
CCGTTGGGA(TCCCAACGG)	5.5	3.452	0.684	5	5	MYBCORE(CNGTTR) MYB2(YAACKG)
GAGTAGCCCA(TGGGCTACTC)	7.6	3.329	0.674	4	2	SITEIIATCYTC(TGGGCY)
TAGCCGTTAC(GTAACGGCTA)	13.3	9.412	0.654	10	10	MYBCORE(CNGTTR) MYB2(YAACKG)
GTAACGGCTA(TAGCCGTTAC)	6.5	9.412	0.654	5	5	MYBCORE(CNGTTR) MYB2(YAACKG)
GACCGTTGC(GCAACGGTC)	10	4.341	0.654	9	9	MYBCORE(CNGTTR) MYB2(YAACKG)
GCTTCGAGTA(TACTCGAAGC)	5.2	3.697	0.648	4	4	
GTCCGGTTG(CAACCGGAC)	4.4	3.327	0.644	4	4	MYBCORE(CNGTTR)
CGGCTAGTTT(AAACTAGCCG)	8.2	5.418	0.643	6	6	
GAGGCGCCAA(TTGGCGCCTC)	11.6	3.768	0.633	4	4	E2F
AGACTAGCCG(CGGCTAGTCT)	7.6	5.03	0.629	4	4	
CGATCCGCGT(ACGCGGATCG)	11.4	5.865	0.609	4	4	OCTAMERMOTIFTAH3H4(CGCGGATC)
CAACGGCTAG(CTAGCCGTTG)	15.8	9.329	0.605	8	8	MYBCORE(CNGTTR) MYB2(YAACKG)
CTAGCCGTTG(CAACGGCTAG)	12.1	9.329	0.605	6	6	MYBCORE(CNGTTR) MYB2(YAACKG)
AACGGCTAC(GTAGCCGTT)	6.7	3.983	0.605	10	9	
CACCTCCCTC(GAGGGAGGTG)	12.4	4.077	0.604	5	5	
AACGGTCGAA(TTCGACCGTT)	12.1	6.466	0.601	9	9	
TGAGTCCCAT(ATGGGACTCA)	4.9	3.168	0.597	4	4	
AACGGCTATA(TATAGCCGTT)	3.8	4.099	0.592	5	5	
CAACGGTCAT(ATGACCGTTG)	7.6	4.514	0.564	6	6	MYBCORE(CNGTTR) MYB2(YAACKG)
TCGATCCTCG(CGAGGATCGA)	9.5	5.201	0.534	5	5	
TCCCACTAGC(GCTAGTGGGA)	7	3.205	0.515	4	4	
AGAGACCGAA(TTCGGTCTCT)	6.3	3.308	0.507	5	5	
GATTCCACTT(AAGTGGAATC)	4.6	3.04	0.506	6	6	
CAACGGTCAC(GTGACCGTTG)	15	3.425	0.502	8	8	MYBCORE(CNGTTR) MYB2(YAACKG)
TGGAGCCACG(CGTGGCTCCA)	11.8	3.685	0.492	4	4	SORLIP1AT(GCCAC)
AGCCGTTACA(TGTAACGGCT)	10.3	5.825	0.488	8	8	MYBCORE(CNGTTR) MYB2(YAACKG)
AGTCTCTCCG(CGGAGAGACT)	7.5	3.366	0.481	4	4	
TTGACGGCTC(GAGCCGTCAA)	7.9	3.199	0.462	4	4	WBOXATNPR1(TTGAC)
CTCACCAACC(GGTTGGTGAG)	10	3.426	0.455	6	6	
CGGCTAGT(ACTAGCCG)	4.9	7.158	0.451	14	14	
AGGCGCCAAA(TTTGGCGCCT)	9.5	4.384	0.438	5	5	E2F
TAACGGCTCT(AGAGCCGTTA)	4.9	5.532	0.426	4	4	MYBCORE(CNGTTR) MYB2(YAACKG)
TCTCAACGGT(ACCGTTGAGA)	4.9	3.998	0.425	4	4	MYBCORE(CNGTTR) MYB2(YAACKG)
CGTAACGGC(GCCGTTACG)	5.1	3.522	0.408	5	5	MYBCORE(CNGTTR) MYB2(YAACKG)
GACCGTTGAG(CTCAACGGTC)	10.3	4.928	0.406	5	5	MYBCORE(CNGTTR) MYB2(YAACKG)
CCGTTGAGA(TCTCAACGG)	7.3	4.384	0.399	10	10	MYBCORE(CNGTTR) MYB2(YAACKG)
AACGGTCGTC(GACGACCGTT)	7.8	5.234	0.398	4	4	
CGACCGTTG(CAACGGTCG)	10	7.715	0.387	9	9	MYBCORE(CNGTTR) MYB2(YAACKG)
ACGATCCGGT(ACCGGATCGT)	7.8	3.386	0.374	4	4	
AAAGGAGGCC(GGCCTCCTTT)	7.9	3.896	0.373	4	3	
CAACGGTCGA(TCGACCGTTG)	17.8	5.962	0.372	9	9	MYBCORE(CNGTTR) MYB2(YAACKG)
GGCATGGATA(TATCCATGCC)	5.4	3.746	0.372	4	4	
ATGACCGTT(AACGGTCAT)	6.3	5.867	0.368	14	14	
TAATCAACGG(CCGTTGATTA)	4.8	3.454	0.367	6	6	MYBCORE(CNGTTR) MYB2(YAACKG) ARR1AT(NGATT)
CACTGCACAC(GTGTGCAGTG)	6.9	3.792	0.351	4	4	
GCCGTTAACG(CGTTAACGGC)	7.8	3.444	0.35	4	4	MYBCORE(CNGTTR) MYB2(YAACKG)
GACCGTTG(CAACGGTC)	12.9	8.74	0.342	30	28	MYBCORE(CNGTTR) MYB2(YAACKG)
CTCACAACAC(GTGTTGTGAG)	9.1	3.504	0.342	8	6	RAV1AAT(CAACA)
AGCCGTT(AACGGCT)	8.9	8.567	0.334	72	62	

Table 6: Putative motifs of Arabidopsis cell-cycle genes with Z_g-score greater than 3.0 and G-score greater than 0.2. **#Occ.** and **#Pr.** are the number of occurrences of a motif and the number of promoters containing the motif. *Continued from Table 5.*

**Motif (complimentary)**	Z**-score**	Z_g**-score**	G**-score**	**#Occ.**	**#Pr.**	**Known motifs**
GTCTCGTTTC(GAAACGAGAC)	6.5	3.054	0.332	5	5	
TTCACCTACC(GGTAGGTGAA)	4.3	4.162	0.33	4	4	
TGACCGTTA(TAACGGTCA)	5.2	4.471	0.329	12	12	MYBCORE(CNGTTR) MYB2(YAACKG)
ACCGTTGGG(CCCAACGGT)	5.5	3.606	0.329	5	5	MYBCORE(CNGTTR) MYB2(YAACKG)
CCAACGGTC(GACCGTTGG)	19.6	5.914	0.328	18	17	MYBCORE(CNGTTR) MYB2(YAACKG)
TCCAACGGTC(GACCGTTGGA)	25	4.921	0.327	13	12	MYBCORE(CNGTTR) MYB2(YAACKG)
AACGGTCA(TGACCGTT)	8.1	6.486	0.325	34	33	
TGACCGTT(AACGGTCA)	6.8	6.486	0.325	29	29	
AATTAGCAGC(GCTGCTAATT)	3.8	3.5	0.323	5	5	
CGACCGTT(AACGGTCG)	5.9	7.609	0.322	17	17	
TTGGAAAGGG(CCCTTTCCAA)	5.7	3.617	0.321	4	3	
AACGGTCAC(GTGACCGTT)	11.5	5.26	0.316	16	16	
AACCGTCGAT(ATCGACGGTT)	8.9	3.879	0.316	7	7	HEXAMERATH4(CCGTCG)
TCACTCTGTA(TACAGAGTGA)	3.7	3.014	0.316	5	5	
GTAGGTGAAG(CTTCACCTAC)	5.7	4.097	0.308	4	4	
TAGAAGAGTG(CACTCTTCTA)	5.4	3.236	0.305	6	6	
GACCGTT(AACGGTC)	8.7	9.404	0.291	71	65	
CCAACGGCT(AGCCGTTGG)	12.7	4.589	0.286	12	12	MYBCORE(CNGTTR) MYB2(YAACKG)
AGCCGTTGG(CCAACGGCT)	8.1	4.589	0.286	7	7	MYBCORE(CNGTTR) MYB2(YAACKG)
TCGATTTTCG(CGAAAATCGA)	4.1	3.002	0.286	5	5	ARR1AT(NGATT)
GTCATCGATC(GATCGATGAC)	10.5	5.645	0.275	8	8	
CCGCTCCTCC(GGAGGAGCGG)	18.8	3.872	0.275	5	5	
TGACCGTTAG(CTAACGGTCA)	5.2	5.995	0.274	4	4	MYBCORE(CNGTTR) MYB2(YAACKG)
CACCAACCCC(GGGGTTGGTG)	14.5	4.095	0.273	6	5	
AACGGTCACA(TGTGACCGTT)	7.4	3.263	0.273	6	6	
ACCACATGGC(GCCATGTGGT)	9.2	3.596	0.272	5	5	MYCATRD22(CACATG) MYCATERD1(CATGTG)
AAACCGTCGA(TCGACGGTTT)	7.4	3.793	0.268	6	6	HEXAMERATH4(CCGTCG)
AGCTGTCTCA(TGAGACAGCT)	6.3	3.635	0.268	5	5	ARFAT(TGTCTC)
ACCGTTG(CAACGGT)	8.3	6.204	0.255	69	63	MYBCORE(CNGTTR) MYB2(YAACKG)
GGCGCCA(TGGCGCC)	5.6	3.006	0.255	20	20	E2F
TGGCGCC(GGCGCCA)	4.5	3.006	0.255	17	16	E2F
AGAATGGCGG(CCGCCATTCT)	8.5	4.779	0.252	4	4	
TTTATGTCAC(GTGACATAAA)	3.9	3.73	0.246	8	7	
CGTAAATGCC(GGCATTTACG)	4.8	4.284	0.243	4	4	
ACCGTTAG(CTAACGGT)	4	3.581	0.243	21	21	MYBCORE(CNGTTR) MYB2(YAACKG)
ACCACGTGA(TCACGTGGT)	5.9	4.188	0.239	9	9	MYC(CANNTG) ABRELATERD1(ACGTG)
TCACGTGGT(ACCACGTGA)	4	4.188	0.239	6	6	MYC(CANNTG) ABRELATERD1(ACGTG)
CCAACGGTCG(CGACCGTTGG)	19.9	6.277	0.236	7	7	MYBCORE(CNGTTR) MYB2(YAACKG)
CAACGGTCCA(TGGACCGTTG)	13.1	4.368	0.236	7	6	MYBCORE(CNGTTR) MYB2(YAACKG)
GAAGCCACAG(CTGTGGCTTC)	7.5	3.185	0.236	4	4	SORLIP1AT(GCCAC)
CTCAACGGCT(AGCCGTTGAG)	7.4	4.906	0.234	4	4	MYBCORE(CNGTTR) MYB2(YAACKG)
CTTAACGGCT(AGCCGTTAAG)	4.9	3.623	0.232	4	4	MYBCORE(CNGTTR) MYB2(YAACKG)
TCAACGGTC(GACCGTTGA)	10.1	4.612	0.23	14	14	MYBCORE(CNGTTR) MYB2(YAACKG)
AATCTCGCCG(CGGCGAGATT)	7.4	3.996	0.228	4	4	ARR1AT(NGATT)
CAACGGC(GCCGTTG)	9.8	4.088	0.226	50	49	MYBCORE(CNGTTR) MYB2(YAACKG)
CCGTTAC(GTAACGG)	5.2	5.874	0.222	57	54	MYBCORE(CNGTTR) MYB2(YAACKG)
AATGTGACTG(CAGTCACATT)	4.2	3.17	0.221	5	5	
ACCGTTGG(CCAACGGT)	7.4	4.717	0.22	19	19	MYBCORE(CNGTTR) MYB2(YAACKG)
GGAAAATCAA(TTGATTTTCC)	7.2	3.399	0.22	13	13	ARR1AT(NGATT)
TCGCGCCAA(TTGGCGCGA)	4.8	3.141	0.22	5	5	
AAGCCTCAGC(GCTGAGGCTT)	9.2	5.313	0.215	5	5	
CAGCCTCATC(GATGAGGCTG)	8.8	4.323	0.214	5	5	
GGACCCCAA(TTGGGGTCC)	5.8	3.138	0.214	6	6	
GATTTTACCC(GGGTAAAATC)	3.7	3.269	0.211	5	5	
CGTTGAGAT(ATCTCAACG)	4.4	3.184	0.209	10	10	
CATGGGCAG(CTGCCCATG)	4.2	3.787	0.207	4	4	
ACAGCGTTTC(GAAACGCTGT)	4.9	3.203	0.202	4	4	
TTGGATTTCA(TGAAATCCAA)	5.7	3.082	0.202	10	10	ARR1AT(NGATT)

Table 7: Discovered Arabidopsis cell-cycle related motifs after word clustering. We clustered 110 words in table 5 and 6 into 55 clusters based on the sequence similarity. In each cluster, motifs overlap at least 7 nucleotides. For each cluster, we plot the motif logo based on their target sites in all the cell-cycle genes. The whole table is in the excel file WangSuppTable7.xls.
